# Do Women Have a Seat at the Table: Trends in Female Representation Among the Board of Directors in American Urological Association Subspecialty Societies

**DOI:** 10.7759/cureus.22502

**Published:** 2022-02-22

**Authors:** Alexandra D Dullea, Daniel C Gonzalez, Rohit Reddy, Parris Diaz, Isaac Zucker, Jessica Delgado, Sirpi Nackeeran, Ruben Blachmann-Braun, Logan Jones, Aditya Sathe, Neha Reddy, Laura Martin, Raveen Syan, Ranjith Ramasamy

**Affiliations:** 1 Urology, University of Miami, Miami, USA; 2 Medicine, Florida International University, Miami, USA; 3 Urology, Jackson Memorial Hospital, Miami, USA

**Keywords:** representation, leadership, aua, disparities, gender

## Abstract

Introduction

Although women remain vastly underrepresented in urology, the proportion of female urology residents and practicing urologists has steadily increased over the last four decades. However, it remains critical to evaluate the representation of females in the pipeline when examining trainees and practicing urologists. As it pertains to leadership positions, the gender distribution among the board of directors (BOD) and committee chairs in the American Urological Association (AUA) subspecialties has not been studied to date. Therefore, we plan to analyze the proportion of females among the BOD and committee chairs in different subspecialty societies recognized by the AUA over time.

Methods

We conducted a cross-sectional observational study, quantitatively comparing the composition of gender in BOD and Committee Chair members belonging to different AUA-recognized subspecialty societies from 2014 to 2020. The websites for each subspecialty society were searched and contacted.

Results

We evaluated BODs from 10 AUA subspecialty societies and committee chair members from 6 AUA subspecialty societies. From 2014 to 2020, the total proportion of female BOD amongst all AUA sub-specialty societies did not change significantly, with a small increase from 10.6% (n = 29) to 13.5% (n = 36). However, female representation among committee chair members significantly increased from 9.8% (n = 20) to 19.2% (n = 44; p = 0.006), along with the total number of women in urology, from 897 (8.9%) to 1,375 (10.3%). Increases in female representation were seen in the Society for the Study of Male Reproduction (SSMR) from 0% to 9% and in the Indian American Urological Association (IAUA) from 4% to 13%. Of note, there were no elected female board members in the Society of Urologic Oncology (SUO) or the Urologic Society for Transplantation and Renal Surgery (USTRS) from 2014 to 2020.

Conclusion

Females remain a minority in leadership positions at AUA sub-specialty societies despite increased female representation in recent years. Future efforts should promote the advancement of women to positions of leadership to reflect the changing landscape of the urology workforce and surgical specialties.

## Introduction

Although women remain vastly underrepresented in urology, the proportion of female urology residents and practicing urologists has steadily increased over the last four decades. Based on the American Urological Association (AUA) census data, women accounted for 10.3% of all practicing urologists in 2020, compared to 7.3% in 2014. As of 2020, there are only 570 female urologists in academic institutions, representing 14.8% of all academic urologists in the US [1]. Although the field has seen upwards of a 30% increase in practicing female urologists since 2014, it is critical to evaluate the representation of females in the pipeline when examining trainees and practicing urologists. Statistics from the 2021 urology residency match revealed that 29.7% of urology applicants were female and that the number of female applicants continues to increase each year [1,2]. While this trend indicates that demographics of the future urology workforce are rapidly shifting, females remain underrepresented in urology and its respective subspecialties with potential barriers in academia [3].

Ideally, the changing landscape of gender demographics within the urologic workforce should reflect a proportionate increase in the number of women in positions of leadership. Urology subspecialties such as andrology/infertility and oncology have historically been male-dominated, while both female pelvic and pediatric urology have greater (though still a minority) female representation [3]. Given the sociological principle that female representation in leadership begets increased female participation at lower levels, we sought to investigate the leadership demographics in different subspecialties [4]. While men still comprise 90% of the urology workforce as of 2019, studies show female urologists disproportionately manage female urologic concerns. In addition to reasons of patient preferences, referral patterns, and subspecialty training, it is very possible that females have higher loads and expectations as practicing urologists [5].

As it pertains to leadership positions, the gender distribution among the board of directors (BOD) and committee chairs in AUA subspecialties has not been studied to date. With the significant shift towards growing female representation in urology, where about a third of incoming urology residents and nearly a quarter of urologists younger than 45 years old are female, we aimed to better define the current landscape of women in urology [2]. In addition, we characterized and analyzed the proportion of females among BOD and committee chairs in different subspecialty societies recognized by the AUA over time.

## Materials and methods

From 2014 to 2020, we conducted a cross-sectional observational study of BOD and committee chair members in different subspecialty societies recognized by the AUA that participated in the annual AUA meeting. If unavailable online, data were requested from the respective society's administration. Of the 15 subspecialty societies, 10 were selected for review as they had complete data sets: Society for Basic Urologic Research (SBUR), Society for Urodynamics, Female Pelvic Medicine and Urogenital Reconstruction (SUFU), Research on Calculus Kinetics Society (ROCK), Engineering and Urology Society (E&U), Indian American Urological Association (IAUA), Society of Academic Urologists (SAU), Society for the Study of Male Reproduction (SSMR), Sexual Medicine Society of North America (SMSNA), Society of Urologic Oncology (SUO), and Urologic Society for Transplantation and Renal Surgery (USTRS). The websites for each subspecialty society were queried between 2014 and 2020 for a full listing of BOD and Committee Chair members. The total number of BOD and Committee Chair members was recorded. Institutional Review Board approval was waived by the University of Miami Institutional Review Board.

A complete list of BOD members was available for 10 out of 15 societies, and 6 out of 15 societies for committee chair positions. In order to assign gender, traditional naming conventions were utilized [6,7]. Web searches via Google were performed for photos in cases of gender-ambiguous names. All BOD members available were accounted for in the denominator. The comparison group used was the percentage of practicing female urologists within each subspecialty in the US based on the AUA census data from 2018. Transgender urologists were not accessed in the analysis as many of the societies do not report numbers in their respective data. Statistical analysis included calculating the total percentage of female BOD and Committee Chair members for each subspecialty association each year and the total proportion of females from 2014 to 2020. Results are published in two or even three-year intervals to assess change evenly over the six years. Statistical analysis was performed with SPSS version 24 software (IBM Corp., Armonk, NY). Categorical variables were analyzed with a Chi-square or Fischer's exact test as required. A p-value < 0.05 was determined to be statistically significant.

## Results

**Figure 1:**
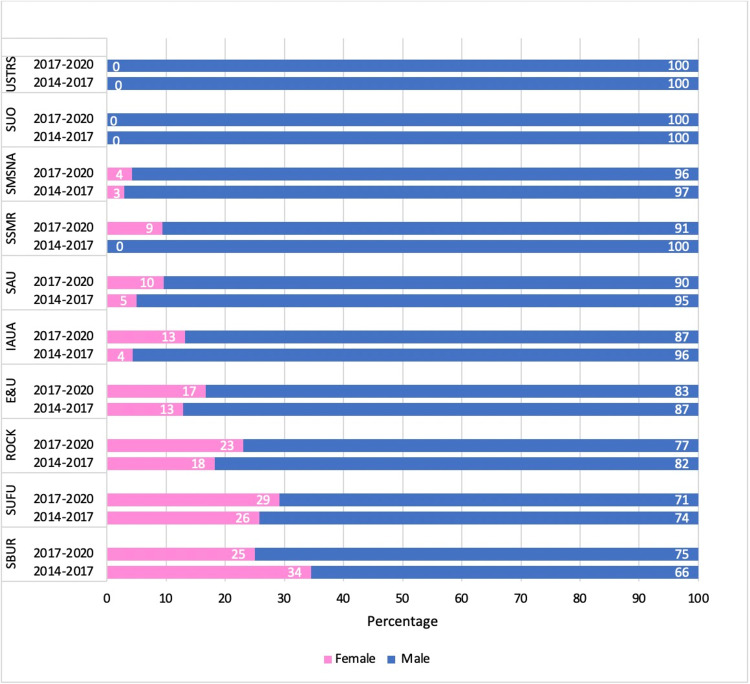
Gender distribution in board of directors of AUA sub-specialty societies between 2014-2017 and 2017-2020. USTRS: Urologic Society for Transplantation and Renal Surgery, SUO: Society of Urologic Oncology, SMSNA: Sexual Medicine Society of North America, SSMR: Society for the Study of Male Reproduction, SAU: Society of Academic Urologists, IAUA: Indian American Urological Association, E&U: Engineering and Urology Society, ROCK: Research on Calculus Kinetics Society, SUFU: Female Pelvic Medicine and Urogenital Reconstruction, SBUR: Society for Basic Urologic Research.

A total of 10 AUA sub-specialty societies' BODs were reviewed from 2014 to 2020, totaling 540 members. Among the AUA sub-specialty BODs in the 2014-2017 period, SBUR demonstrated the highest proportion of females with 10 (34.5%), followed by SUFU with 9 (25.7%). In the 2014-2017 period, there was no female BOD representation within SSMR, SUO, and USTRS subspecialty societies. Between 2017 and 2020, the highest proportion of females was observed in SUFU with 7 (29.2%), followed by SBUR with 9 (25%). There was no female representation in the SUO and USTRS subspecialty societies. The greatest increases in female representation were seen in the SSMR (0% to 9%) and IAUA (4% to 13%). However, the proportion of female BOD members declined over time in SBUR (34% to 25%; Figure 1). Over this time period, the total proportion of female BOD amongst all AUA sub-specialty societies increased from 29 (10.6%) to 36 (13.5%) (p = 0.292; Table 1).

**Table 1:** Comparison of the gender distribution between 2014-2017 and 2017-2020 of the AUA sub-specialty societies BOD. SBUR: Society for Basic Urologic Research, SUFU: female pelvic medicine and urogenital reconstruction, ROCK: Research on Calculus Kinetics Society, E&U: Engineering and Urology Society, IAUA: Indian American Urological Association, SAU: Society of Academic Urologists, SSMR: Society for the Study of Male Reproduction, SMSNA: Sexual Medicine Society of North America, SUO: Society of Urologic Oncology, USTRS: Urologic Society for Transplantation and Renal Surgery, AUA: American Urological Association, BOD: board of directors.

	Period	Total members	Female	Male	P-value
SBUR	2014-2017	29	10 (34.5%)	19 (65.5%)	
	2017-2020	36	9 (25%)	27 (75%)	0.403
SUFU	2014-2017	35	9 (25.7%)	26 (74.3%)	
	2017-2020	24	7 (29.2%)	17 (70.8%)	0.770
ROCK	2014-2017	11	2 (18.2%)	9 (81.8%)	
	2017-2020	13	3 (23.1%)	10 (76.9%)	0.769
E&U	2014-2017	31	4 (12.9%)	27 (87.1%)	
	2017-2020	24	4 (16.7%)	20 (83.3%)	0.695
IAUA	2014-2017	23	1 (4.3%)	22 (95.7%)	
	2017-2020	38	5 (13.2%)	33 (86.8%)	0.263
SAU	2014-2017	40	2 (5%)	38 (95%)	
	2017-2020	42	4 (9.5%)	38 (90.5%)	0.432
SSMR	2014-2017	39	0	39 (100%)	
	2017-2020	32	3 (9.4%)	29 (90.6%)	0.087
SMSNA	2014-2017	35	1 (2.9%)	34 (97.1%)	
	2017-2020	24	1 (4.2%)	23 (95.8%)	0.785
SUO	2014-2017	20	0	20 (100%)	
	2017-2020	18	0	18 (100%)	0.999
USTRS	2014-2017	11	0	11 (100%)	
	2017-2020	15	0	15 (100%)	0.999
Overall AUA sub-specialty societies BOD	2014-2017	274	29 (10.6%)	245 (89.4%)	
	2017-2020	266	36 (13.5%)	230 (86.5%)	0.292

**Figure 2:**
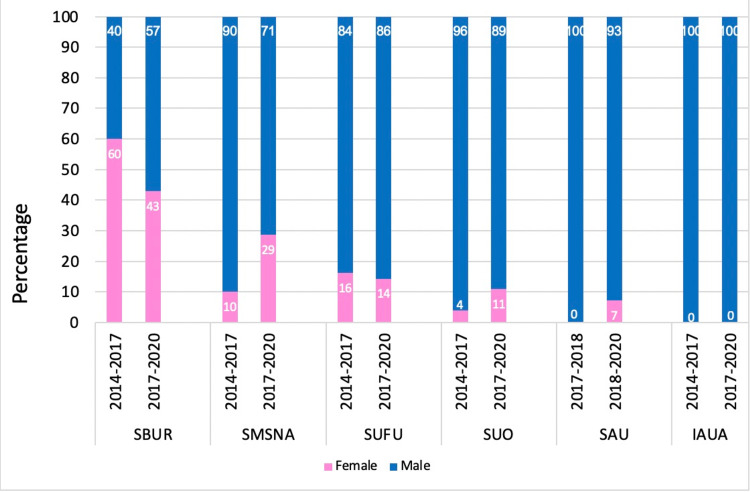
Gender distribution in committee chair positions of AUA sub-specialty societies between 2014-2017 and 2017-2020. SBUR: Society for Basic Urologic Research, SMSNA: Sexual Medicine Society of North America, SUFU: female pelvic medicine and urogenital reconstruction, SUO: Society of Urologic Oncology, SAU: Society of Academic Urologists, IAUA: Indian American Urological Association.

A total of six individual AUA Committees were reviewed between 2014 and 2020, which included 433 chair members. Among the AUA Committee Chair members in the 2014-2017 period, the highest proportion of females was in the SBUR with 9 (60%), followed by SUFU with 5 (16.1%). There was no female representation in the SAU and IAUA. In the 2017-2020 period, the highest percentage of females was in the SBUR with 42.9% (n = 18), followed by the SMSNA with 28.6% (n = 10). From 2014 to 2020, there was no female representation in the IAUA. The greatest increases in female representation were seen in the SUO (3.9% to 10.8%) and SMSNA (10% to 28.6%). However, the proportion of female committee chairs declined over time in SBUR (60% to 42.9%; Figure 2). Over this time course, the total proportion of female AUA Committee Chair members increased significantly from 20 (9.8%) to 44 (19.2%) (p=0.006; Table 2).

**Table 2:** Comparison of the gender distribution between 2014-2017 and 2017-2020 of the AUA Committee Chair members. SBUR: Society for Basic Urologic Research, SMSNA: Sexual Medicine Society of North America, SUFU: female pelvic medicine and urogenital reconstruction, SUO: Society of Urologic Oncology, SAU: Society of Academic Urologists, IAUA: Indian American Urological Association, AUA: American Urological Association.

	Period	Total members	Female	Male	P-value
SBUR	2014-2017	15	9 (60%)	6 (40%)	
	2017-2020	42	18 (42.9%)	24 (57.1%)	0.254
SMSNA	2014-2017	20	2 (10%)	18 (90%)	
	2017-2020	35	10 (28.6%)	25 (71.4%)	0.176
SUFU	2014-2017	31	5 (16.1%)	26 (83.9%)	
	2017-2020	35	5 (14.3%)	30 (85.7%)	0.550
SUO	2014-2017	102	4 (3.9%)	98 (96.1%)	
	2017-2020	83	9 (10.8%)	74 (89.2%)	0.067
SAU	2014-2017	16	0	16 (100%)	
	2017-2020	28	2 (7.1%)	26 (92.9%)	0.526
IAUA	2014-2017	20	0	20 (100%)	
	2017-2020	6	0	6 (100%)	0.999
Overall AUA Committee Chair members	2014-2017	204	20 (9.8%)	184 (90.2%)	
	2017-2020	229	44 (19.2%)	185 (80.8%)	0.006

## Discussion

This study highlights the current landscape of women in leadership positions within different AUA subspecialties, ranging from BOD to Committee Chairs. Our investigation revealed that females consistently represented a minority of elected BOD and Committee chairs within each subspecialty throughout 2014 to 2020 (Figure 1). However, the total proportion of female BOD and committee chair members increased over time.

The trends in gender composition among BOD and committee chair positions in subspecialty societies were unknown prior to this study. Our results confirm that certain urologic subspecialties were associated with higher female representation in academic leadership positions over time. Notably, the Society for Urodynamics, Female Pelvic Medicine, and Urogenital Reconstruction (SUFU) consistently exhibited the second-highest female representation throughout 2014-2020 in both BOD and Committee Chair positions. Conversely, our study identified societies with notable gender disparities. From 2014-2020, groups such as the SUO and the USTRS did not have any elected female board members. Similar outcomes are observed following a broader study that analyzed the trends in gender distribution among urologists after entering the workforce via American Board of Urology (ABU) certification or recertification logs. Nettey et al. found that women reported subspecializing in female urology (24.2%) and pediatric urology (10.2%) at higher frequencies when compared to their male colleagues (4.6% and 3.1%, respectively) [8]. In contrast, men identified as oncologists and endocrinologists 1.7 times and 1.3 times more frequently than their female colleagues. The data imply possible preconceived notions regarding gender roles in urology sub-specialization and job selection. It is of utmost importance to examine these gender disparities, as they affect the urologic workforce and are associated with differences in compensation, career development, grant rewards, and authorship within academic medicine [9].

The 2017-2018 academic year marked the first year where women outnumbered men in medical school enrollees [10], further emphasizing the importance of examining the specialties with lower female representation, identifying barriers to gender parity, and proposing efforts to close the gender gap [11]. Currently, women comprise only 10% of practicing urologists. However, when compared to other specialties, urology has demonstrated nearly a 30-fold increase in female residents since 1978 [12,13]. Exceeding the overall match rate for the first time since 2014, females in the 2020 AUA Match achieved a record match rate of 86%. Promoting and retaining women in academic urology may ultimately trickle down to the medical school level and positively impact female medical students pursuing urology.

While current trends have demonstrated progress in narrowing the gender gap, women remain considerably underrepresented in leadership roles within academic urology. Although women encompassed only 3.3% of the department and/or division chairs and 8.1% of program directors in 2016, a recent study demonstrated a two-fold increase for both positions in 2020 [2,14]. Another recent study analyzed the trends of female leadership through speakership at major urologic conferences. Females consistently represented a minority of speakers at every conference evaluated. However, the total proportion of female speakers increased from 13.7% in 2014 to 19.3% in 2019 (p=0.005) [15]. Overall, the data demonstrate that gender gaps are emphasized in academic leadership positions. These discrepancies may reflect the lack of mentorship and professional guidance within the urologic pipeline. Evidence has shown that the proportion of female residents within a urology residency training program is positively correlated with the number of female faculty [2]. Therefore, the increasing proportion of female representation in academic leadership positions may explain the increase in female urology applicants. Current gender disparities necessitate effective mentorship to continue increasing female exposure to urologic subspecialties and equal representation in academic leadership positions.

Our study has inherent limitations for this type of observational analysis, which may make it difficult to identify correlations. Although we examined each society’s elected BOD and committee chairs, we did not examine each society’s overall committee and membership composition. This may be useful because transparency in the gender composition of specialty societies is an important baseline to establish. In addition, we were unable to quantify the number of female candidates in the applicant pool for these society positions. Moreover, some societies may rely on the advancement of leadership positions, leading to the possibility of lead-time bias in our results. Furthermore, despite contacting all 16 subspecialty societies, we only received data from 10 societies, and thus do not know if other societies reflect similar opportunities for women. Finally, we were unable to determine transgender representation among the various urological subspecialty societies as this is not normally reported, thereby leading to the unfortunate exclusion of individuals who have also traditionally faced gender discrimination and underrepresentation. Despite these limitations, our study gives insight into the current climate of women in positions of leadership. To the best of our knowledge, this study is the first to report on gender differences among positions of leadership in AUA subspecialty societies. Future studies should be aimed at examining the contributing factors that influence residents’ decisions to pursue the field. Further research is necessary to determine whether the higher percentage of women in leadership roles in urologic societies correlates with an increase in women joining the subspecialty and society.

## Conclusions

The number and proportion of women in BOD and committee chair positions are increasing overall among the various AUA subspecialty societies. However, there are still significant disparities in various subspecialties. The first step toward diversity and inclusion, and ultimately equity, is transparency. We ultimately hope that this information will inform societies as they consider ways to include more women in positions of leadership. Societies should evaluate the processes by which they recruit for BOD and committee chair positions and, ideally, focus on improving disparities that exist. Additionally, as women in urologic residency choose potential fellowship paths, facts about gender composition in the various subspecialties may be useful.
